# Content development for a physical activity and sedentary behaviour e-learning module for early childhood education students: a Delphi study

**DOI:** 10.1186/s12889-020-09670-w

**Published:** 2020-10-23

**Authors:** Brianne A. Bruijns, Andrew M. Johnson, Patricia Tucker

**Affiliations:** 1 Health and Rehabilitation Sciences Program, Faculty of Health Sciences, University of Western Ontario, London, ON Canada; 2 School of Health Studies, Faculty of Health Sciences, University of Western Ontario, London, ON Canada; 3 School of Occupational Therapy, Faculty of Health Sciences, University of Western Ontario, 1201 Western Road, Elborn College, Room 2547, London, ON N6G 1H1 Canada

**Keywords:** Physical activity, Sedentary behaviour, Screen-viewing, Early childhood education, Young children, E-learning

## Abstract

**Background:**

Early childhood educators play a prominent role in promoting healthy activity behaviours in childcare. However, they have expressed that they lack the appropriate pre-service training to confidently lead physical activity (PA), and minimize sedentary behaviour (SB), in childcare. As such, the purpose of this Delphi study was to generate and reach agreement on content areas for inclusion in a PA and SB e-Learning module for Early Childhood Education (ECE) students.

**Methods:**

Purposeful sampling of Canadian/international researchers was used to form two expert panels: a PA/SB expert panel (*n* = 26), and a Canadian ECE panel (*n* = 35). The PA/SB experts suggested their top 12 PA/SB topics for the module via online survey. These topics were then pooled to generate a list of 19 content areas. In a second online survey, both panels of experts rated the importance of each content area (0 = unimportant to 5 = very important). Mean ratings (*M*) were ranked separately for each panel, and then ratings were pooled to create an overall ranking of the 19 content areas. Inter-panel agreement of importance rankings was visually represented as a scatterplot and quantified using Spearman’s rho (*r*_*s*_).

**Results:**

The top-rated content area was *Outdoor Play* (*M* = 4.77 ± 0.64), followed by *Benefits of PA in the Early Years* (*M* = 4.75 ± 0.66)*,* and *Factors Influencing PA and SB in Childcare* (*M* = 4.71 ± .74)*. Monitor PA and Sedentary Time Within Your Classroom* had the lowest combined score (*M* = 3.77 ± 1.44). There was moderate-to strong inter-panel agreement for content area importance rankings (*r*_*s*_ = 0.60, 95% CI: 0.20 to 0.83). The majority of the ECE expert panel considered this training important for ECE students (94.3%), aligning with ECE curriculum objectives (91.4%) and accreditation standards (78.8%), and complementary to the present ECE curriculum (88.6%).

**Conclusions:**

Providing PA and SB training to ECE students is a proactive way to ensure healthy movement behaviours are prioritized in childcare programming. With the PA/SB expert-developed content areas, and endorsement by the ECE expert panel, implementing this training within ECE programs is a necessary next step.

**Supplementary information:**

**Supplementary information** accompanies this paper at 10.1186/s12889-020-09670-w.

## Background

For young children (< 5 years), regular participation in physical activity is key to healthy physical, psychosocial, and cognitive development [1]. Specifically, increased duration and frequency of physical activity in early childhood positively influences executive function and language, [2] while higher intensity physical activity has been associated with improved motor skill development [3]. Further, limiting prolonged sedentary time, particularly in front of screens, is critical; in young children, television-viewing has been linked to decreased attention and disruptive sleep, [4] as well as decreased cognitive development (including literacy and numeracy) [5]. As such, establishing healthy physical activity and sedentary behaviour habits in early childhood is highly important, and the childcare environment, where two-thirds of young Canadian children spend the majority of their weekdays, [6] has been identified as a prime setting to target these health behaviours.

Early childhood educators are influential role models in the childcare setting, and with respect to programming, they control a substantial portion of young children’s days [7–9]. However, research has shown that both educator values and self-efficacy relating to physical activity, as well as their level of training in this area, influence the amount of physical activity they incorporate in their programming [10–13]. Early childhood educators have acknowledged their limited pre-service training in physical activity and sedentary behaviour, [12, 14] and have associated this with their low self-efficacy to promote and lead physical activity opportunities in childcare [10]. A recent Canadian study found that only 32.2 and 26.7% of Canadian Early Childhood Education (ECE) students reported receiving physical activity and screen-viewing-related training in their post-secondary ECE programs, respectively [15]. Further, in Canada, only 3 provinces/territories specifically reference physical activity, and 1 references screen-viewing, in their childcare regulation, [16] and few childcare centres have adopted physical activity (30%) and screen-viewing (29%) policies of their own [17]. Given such limited regulations, it is often early childhood educators who are responsible for determining the duration and frequency of physical activity opportunities and screen use in their classroom [11, 12]. With the strong curricular focus placed on preparing children in their care for school, educators may not consider opportunities for physical activity as integral programming components [18, 19]. Providing educators with proper training in physical activity has been introduced as a possible solution to ensure children are afforded appropriate daily opportunities to be active [20].

Both early childhood educators [21] and ECE students [22] have expressed their desire for additional training in physical activity and sedentary behaviour, and the provision of such learning opportunities is essential to assist educators in promoting the development of healthy movement behaviours among children in childcare. Recent efforts to better support educators in promoting and leading physical activity, and minimizing excessive sedentary time in childcare environments, have shown promising results [23–26] (Bruijns et al.: Early childhood educators’ physical activity-related self-efficacy and knowledge following the SPACEand SPACE-Extension physical activity interventions in childcare, submitted). For example, interventions that have provided early childhood educators with physical activity training have resulted in preschoolers accumulating increased moderate-to vigorous-intensity physical activity (MVPA; + 0.5 min/day and + 1.28 min/day) [23, 25], and decreased sedentary time (− 2.13 min/day) while in childcare [25]. Early childhood educators’ receptiveness to both of these interventions was positive, and they communicated that they would continue to use the knowledge gained from the training after the interventions ceased [24, 27]. While professional development in physical activity and sedentary behaviour for educators is essential to support ongoing learning and scaffold their physical activity-related teaching self-efficacy, [23] (Bruijns et al.: Early childhood educators’ physical activityrelated self-efficacy and knowledge following the SPACE and SPACE-Extension physical activity interventions in childcare, submitted) there is a need for this supplementary education at the post-secondary level (i.e., within ECE programs). This initiative will ensure ECE graduates are well-prepared to support healthy movement behaviours among young children upon entering a childcare-based profession [28].

Given the success of physical activity training programs for early childhood educators, [23, 25] and the importance of providing this training to all early childhood educators pre-employment (where physical activity-related education is lacking), [15] the next step is to narrow down key physical activity and sedentary behaviour content areas to include in training at the pre-service level. Further, there is a need to introduce more educator outcome measures (e.g., physical activity-related knowledge, self-efficacy, and teaching behaviours) in order to find out what content best supports educators’ knowledge acquisition and retention, as well as their development of self-efficacy to lead physical activity and minimize prolonged sedentary time in childcare. As such, the goal of the **T**raining **EA**rly **CH**ildhood educators in physical activity study (i.e., the TEACH study), is to develop, implement, and evaluate the impact of a physical activity and sedentary behaviour e-Learning module for students in Canadian post-secondary ECE programs. As a first step, the current study aimed to identify and reach agreement on physical activity and sedentary behaviour content areas that are necessary for early childhood educators to be trained in.

## Methods

The Non-Medical Research Ethics Board at the University of Western Ontario provided ethical approval (REB# 114435) for the conduct of this research.

### Study design

The Delphi method, developed by Dalkey and Helmer (1963), was adopted as the study design, as it is appropriate in cases where the subjective opinion of a group of experts is needed to reach consensus on a topic, but these individuals cannot meet to discuss in-person (due to constraints such as distance and time) [29, 30]. The Delphi technique involves multiple rounds of surveys with controlled feedback, allowing participants to reassess their answers based on their review of other panelists’ responses [29]. Further, this method allows for anonymity, which mitigates challenges associated with traditional group consensus methods, where dominant individuals and pressure to conform can be confounding factors [31]. The study design and procedures were loosely modeled after Gillis and colleagues’ [32] Delphi study, which aimed to achieve consensus on research priorities for children’s and adolescents’ physical activity and sedentary behaviours.

### Participants and recruitment

Canadian (*n* = 13) and international (*n* = 18) early years physical activity and sedentary behaviour experts were identified by the research team and invited via email to participate in two online surveys through Qualtrics©. Experts were selected based on: 1. their established research in the field; and, 2. provincial/geographic location (i.e., to ensure appropriate representation within and outside of Canada). Additional experts (*n* = 17), referred to the research team by the initial group of study participants, were then invited as national (*n* = 2) and international (*n* = 15) experts. If no response was received within 2 weeks, a reminder email was circulated. Recruitment took place in October 2019 and a total of 25 physical activity and sedentary behaviour experts agreed to participate prior to the first round of surveys. One additional expert agreed to participate prior to the second round of surveys (53% response rate).

**Fig. 1:**
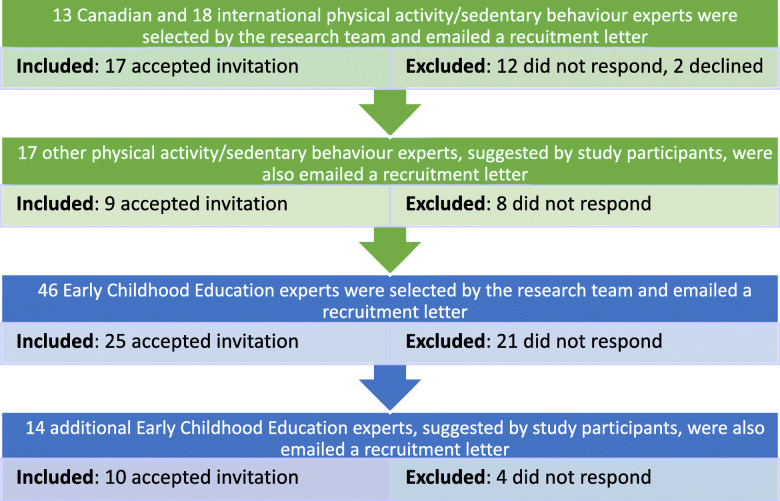
Purposeful sampling process undertaken to recruit physical activity/sedentary behaviour and Early Childhood Education experts

In order to ensure module content was appropriate and contextually relevant to integrate into Canadian ECE curricula, 46 Canadian ECE experts were identified by the research team and invited via email to participate. Experts were selected based on their: 1. occupational position (i.e., ECE university professor, board/executive member of a relevant ECE organization, dean or program head/instructor of a post-secondary ECE program); 2. years of experience in the ECE field (5 years minimum); 3. provincial/territorial location (i.e., to ensure appropriate representation); and, 4. online email address availability. Additional experts (*n* = 14), referred to the research team by the initial group of ECE experts, were also invited to participate. Recruitment took place in November 2019 and a total of 35 ECE experts agreed to participate (58% response rate). See Fig. 1 for the full recruitment process of physical activity/sedentary behaviour and ECE experts.

### Study procedures

Physical activity/sedentary behaviour experts completed two online surveys. The first survey (Additional File 1) gathered their top 12 physical activity and sedentary-behaviour-related content areas they felt should be included in an e-Learning module for ECE students (with a brief justification for each topic). Two study investigators (BAB, PT) reviewed the topics generated in the first round of surveys and pooled them together. Similar topics were merged, and a list of unique content areas was created. Content areas that were only mentioned by one participant were excluded from the final list.

In the second round of surveys, experts were provided the pooled list of content areas (along with a brief description of what would be included in that section of the module). They were asked to rate the importance of each content area on a 5-point Likert scale (0 = unimportant to 5 = very important; Additional File 2). In order to ensure all proposed content areas were captured in the pooled list, experts were asked to indicate whether the topics they proposed in the first survey were accurately represented. Occupational positions for physical activity experts were retrieved by the research team via their institutional websites.

The ECE expert panel completed a version of the second online survey (Additional File 3), which, in addition to gathering their importance ratings of the content areas generated by the physical activity/sedentary behaviour expert panel, also captured: 1. demographics (occupational position, years of experience); 2. suggestions for topics not already proposed; 3. how important they felt this type of training was for ECE students; and, 4. whether they felt the module content aligned with ECE curriculum objectives and accreditation criteria/vocational learning outcomes, and complemented current ECE curriculum.

Experts were assigned a unique participant code to use when filling out each online survey so that study investigators could determine which panel (i.e., Canadian, international, or ECE) each expert belonged to, and who had participated (in order to determine the need for subsequent survey dissemination).

### Data analysis

Descriptive statistics of demographics, content area importance ratings, representation of panel-suggested topics, and perspectives regarding the importance of this type of training were completed in SPSS (version 25). Within each panel of experts (i.e., physical activity/sedentary behaviour and ECE), mean (*M*) scores on each of the 19 content areas was generated. Pearson product-moment correlation coefficient was then calculated between the means of the two panels, and the 19 content areas were ranked within each panel. Similarity in rankings between the two panels was assessed using Spearman’s *rho* (*r*_s_). Analyses were conducted in R version 3.6.1 [33].

## Results

### Demographics

#### Physical activity and sedentary behaviour expert panel

**Fig. 2.**
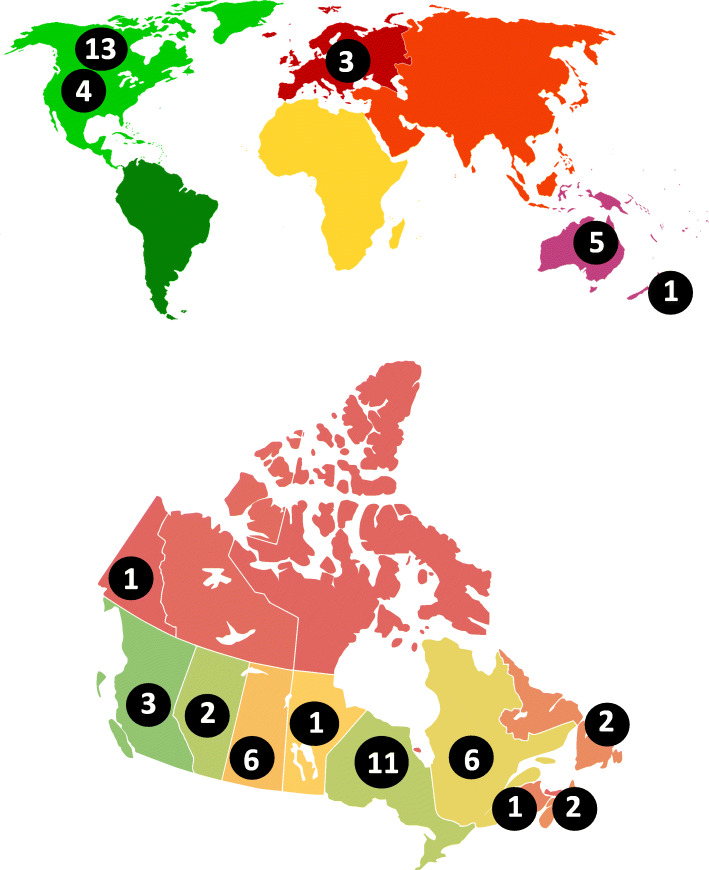
**a** Geographical representation of participating physical activity/sedentary behaviour experts (number indicates how many experts were from that region). **b** Provincial/territorial representation of participating Early Childhood Education experts (number indicates how many experts were from that province/territory). Images retrieved from: https://www.google.com/url?sa=i&url=https%3A%2F%2Fcommons.wikimedia.org%2Fwiki%2FFile%3ABlankMap-World-Continents-Coloured.PNG&psig=AOvVaw1Ol60sFsRvFxutU; https://www.google.com/url?sa=i&url=https%3A%2F%2Fcommons.wikimedia.org%2Fwiki%2FFile%3ACanada_population_per_senat_or_map.svg&psig=AOvVaw304urdoYMCZJkP5QIZKib-&ust=1587728821294000&source=images&cd=vfe&ved=0CAIQjRxqFwoTCJCEgP-8_ugCFQAAAAAdAAAAABAE

Physical activity/sedentary behaviour experts represented 6 different countries (Canada [*n* = 13], Australia [*n* = 5], the United States [*n* = 4], the Netherlands [*n* = 2], the United Kingdom [*n* = 1], and New Zealand [*n* = 1]). All experts held positions in academia (including 2 post-doctoral fellows, 5 assistant professors, 10 associate professors, and 9 full professors). See Fig. 2a for geographical representation of the physical activity/sedentary behaviour expert panel.

#### ECE expert panel

ECE experts represented 10 Canadian provinces/territories (Ontario [*n* = 11], Quebec [*n* = 6], Saskatchewan [*n* = 6], British Columbia [*n* = 3], Alberta [*n* = 2], Newfoundland and Labrador [*n* = 2], Nova Scotia [*n* = 2], Manitoba [*n* = 1], New Brunswick [*n* = 1], and Yukon [*n* = 1]; Fig. 2b). Experts held a wide range of ECE occupational positions, including 6 as university professors (3 assistant professors, 1 associate professor, 1 full professor, and 1 professor emerita), 11 as board/executive members of ECE-related organizations, and 18 as faculty/staff within ECE programs (1 dean, 4 program/department heads/coordinators, 1 curriculum writer, and 12 instructors). On average, these experts had 23.11 ± 11.43^1^ years of experience in the ECE field.

### Physical activity and sedentary behaviour content areas

A total of 22 content areas were generated by the physical activity/sedentary behaviour expert panel. Three content areas were excluded due to not being relevant to other panelists’ topics; as such, 19 content areas were carried forward. The majority (90.5%) of panelists reported their suggested topics were appropriately represented in the final list of content areas. See Supplementary Table 1 for a detailed list and description of the content areas.

**Table 1:** Ranked Content Areas in Physical Activity and Sedentary Behaviour Suggested for Inclusion in an e-Learning Training Module for Canadian Early Childhood Education Students

Content Area	PA/SB Panel (***n*** = 21) ***M*** (***SD***)	PA/SB Panel Rank	ECE Panel (n = 35) ***M*** (***SD***)	ECE Panel Rank	Both Panels ***M (SD)***	Final Rank^a^
Outdoor Play	4.71 (.46)	4	4.83 (.45)	1	4.77 (.64)	**1**
Benefits of Physical Activity in the Early Years	4.81 (.40)	1	4.69 (.53)	3	4.75 (.66)	**2**
Factors Influencing Physical Activity and Sedentary Behaviour in Childcare	4.76 (.44)	2.5	4.66 (.59)	5	4.71 (.74)	**3**
Defining Physical Activity and Sedentary Behaviour	4.57 (.60)	8	4.74 (.44)	2	4.66 (.74)	**4**
Promote Physical Activity and Minimize Sedentary Time through Instruction and Interaction	4.76 (.44)	2.5	4.51 (.66)	8	4.64 (.79)	**5**
Create and Make Use of Environments to be Supportive of Physical Activity	4.57 (.68)	8	^a^4.68 (.48)	4	4.63 (.83)	**6**
Become a Role Model and Champion for Physical Activity	4.62 (.74)	5.5	4.40 (.74)	10.5	4.51 (1.05)	**7**
Program Time for Physical Activity and Active Breaks to Limit Sitting Time	4.62 (.67)	5.5	4.37 (1.03)	12	4.50 (1.23)	**8**
The Canadian 24-Hour Movement Guidelines for the Early Years (< 5 years)	4.38 (.81)	12.5	4.60 (.60)	7	4.49 (1.01)	**9**
Risky Play	4.24 (.83)	15.5	4.63 (.60)	6	4.44 (1.02)	**10**
Get Parents/Guardians On Board!	4.43 (1.03)	10.5	4.40 (.78)	10.5	4.41 (1.29)	**11**
Physical Literacy and Fundamental Movement Skills	4.38 (.81)	12.5	4.43 (.78)	9	4.41 (1.12)	**11**
Incorporate Physical Activity into Other Educational Objectives	4.24 (1.00)	15.5	^a^4.35 (.65)	13	4.30 (1.19)	**13**
Resources and Professional Development	4.33 (.80)	14	4.26 (.78)	15	4.30 (1.12)	**13**
Suggest the Creation of Physical Activity and Screen-Viewing Policies at Your Centre	4.43 (.60)	10.5	^a^4.06 (.92)	16.5	4.25 (1.10)	**15**
Example Activities	4.57 (.75)	8	3.91 (1.10)	18	4.24 (1.33)	**16**
Risks of Excessive Sedentary Behaviour and Screen-Viewing	4.10 (.94)	17	4.34 (.68)	14	4.22 (1.16)	**17**
Prevalence of Physical Activity, Sedentary Behaviour, and Screen-Viewing among Young Children	3.95 (.92)	18	^b^4.06 (.85)	16.5	4.01 (1.25)	**18**
Monitor Physical Activity and Sedentary Time in Your Classroom	3.76 (1.14)	19	3.77 (.88)	19	3.77 (1.44)	**19**

*Note. PA* physical activity, *SB* sedentary behaviour, *ECE* Early Childhood Education, *M* mean, *SD* standard deviation, ^a^ Final rank was determined by the highest combined mean score between panels; ^b^ Only 34 respondents for this question

#### Physical activity and sedentary behaviour expert panel

On average, physical activity/sedentary behaviour experts (*n* = 21^2^) rated all content areas as important to include in the e-learning module (*M* range = 3.76 to 4.81). These experts rated *Benefits of Physical Activity in the Early Years* as the most important content area (*M* = 4.81 ± 0.40), and *Monitor Physical Activity and Sedentary Time in Your Classroom* as the least important content area (*M* = 3.76 ± 1.14]), to include in the e-Learning module. See Table 1 for content area importance rankings.

#### ECE expert panel

ECE experts (*n* = 35) also had moderate to high ratings of the importance of the content areas (*M* range = 3.77 to 4.83). They rated *Outdoor Play* as the most important content area to include in the e-Learning module (*M* = 4.83 ± 0.45]) and *Monitor Physical Activity and Sedentary Time in Your Classroom* as the least important content area (*M* = 3.77 ± 0.88]). See Table 1 for content area importance rankings by panel.

#### Final ranking and inter-panel agreement

**Fig. 3.**
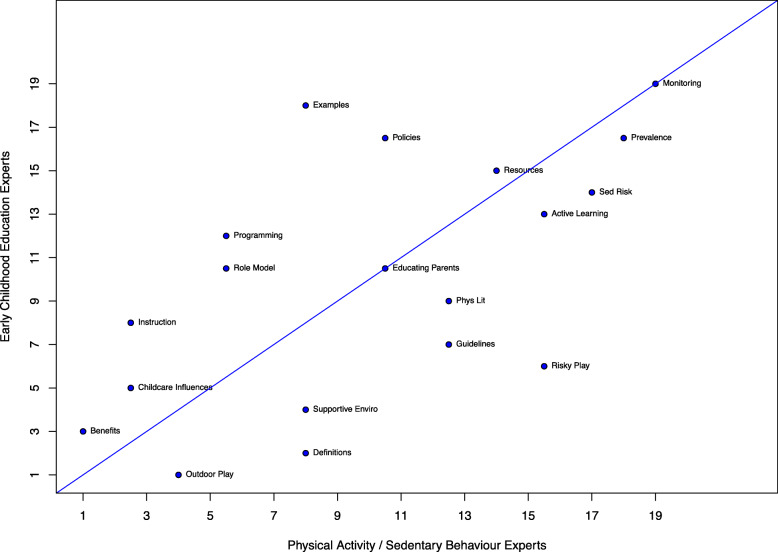
Scatterplot of the associations between panels’ rankings for each content area

In the final ranked list of content areas, *Outdoor Play* (*M* = 4.77 ± 0.64), *Benefits of PA in the Early Years* (*M* = 4.75 ± 0.66), and *Factors Influencing PA and SB in Childcare* (*M* = 4.71 ± 0.74) had the highest combined scores. *Monitor PA and Sedentary Time in Your Classroom* had the lowest combined score (*M* = 3.77 ± 1.44). There was moderate-to-strong inter-panel agreement across the 19 content areas, with mean scores correlating 0.63 (95% CI: 0.25 to 0.84) and ranked scores demonstrating an association (*r*_s_) of 0.60 (95% CI: 0.20 to 0.83). See Fig. 3 for a graphical representation of the associations between panels for each content area ranking.

### ECE panel perspectives on the e-Learning module

The majority of ECE experts (94.3%) rated this type of training as ‘Important’ or ‘Very Important’ for ECE students to receive. Most experts (91.4%) reported they agreed that the physical activity and sedentary behaviour e-Learning module aligned with the objectives of the current post-secondary ECE curriculum, and 88.6% reported they agreed that the training would complement this curriculum. The majority of ECE experts (78.8%) also communicated their agreement that this type of training aligned with ECE professional accreditation standards.

## Discussion

This was the first study to employ the Delphi method to generate physical activity and sedentary behaviour content to be included in training for ECE students. The use of two field-specific expert panels to offer their insights on this training provided a unique perspective on module content development, and their general consensus on important rankings of the content areas provides helpful direction regarding areas of foci for the e-Learning module. A number of important findings from this study are discussed below.

Six content areas proposed by the experts focused on giving ECE students necessary background information on physical activity and sedentary behaviour, ranging from definitions and benefits/risks of these behaviours to guidelines and current prevalence rates. These content areas are essential to include, as ECE students have noted the lack of physical activity and sedentary behaviour-specific training in their program [15]. Bruijns et al. (2019) surveyed 1292 ECE students, and while the majority of students reported that their courses covered gross motor development (86.6%), few covered concepts such as physical literacy (46.2%), screen-viewing (47.3%), or sedentary behaviour (41.5%) [15]. Without a proper introduction to these concepts and their importance to consider when programming, it is unlikely that ECE students will be receptive to strategies to promote physical activity and minimize sedentary time [22]. As evidenced by Bruijns et al. (in-press), ECE students felt it was more important and their responsibility to teach physical activity-related skills (such as fitness activities, locomotor skills, and play skills) in childcare if they reported receiving training in physical activity [22]. As such, if ECE students are introduced to these concepts during their pre-service schooling, it is likely that they will promote healthy movement behaviours among the children they care for upon entering the ECE profession.

Physical activity/sedentary behaviour experts also suggested including training related to factors that influence young children’s physical activity and sedentary behaviour in the childcare environment, with specific attention paid to outdoor and risky play (receiving heightened attention in the ECE field as of late [34]. This review of correlates is critical within the module, as it will highlight to ECE students the varying aspects of the childcare environment, and educator behaviours, that act as facilitators/barriers to children’s physical activity and that influence their sedentary behaviours [13, 35]. Stemming from the review of correlates, eight additional content areas suggested by the panel related to providing ECE students with practical strategies on how to promote physical activity and minimize sedentary time in their classroom (noted as important within childcare educator training interventions) [26, 36]. In addition, two content areas focused on helpful resources and training, and practical video example activities, to further aid ECE students in this respect. The focus of the content suggested for the module on these strategies and resources is encouraging, as educators have reported they lack the appropriate training on how to lead skill-based physical activities in childcare [37]. Further, early childhood educator training interventions have noted the benefit of this type of practical support in scaffolding educators’ physical activity-related self-efficacy, (Bruijns et al.: Early childhood educators’ physical activity-related self-efficacy and knowledge following the SPACE and SPACE-Extension physical activity interventions in childcare, submitted) and both increasing physical activity [38] and decreasing sedentary time [26] among children in their care. Offering video examples may teach ECE students how to engage children in physical activity, and promises to support their self-efficacy in this pursuit via vicarious reinforcement and modeling [39].

While both expert panels expressed their views of the importance of all proposed content areas for the e-Learning module, the top-rated content areas (i.e., *Outdoor Play, Benefits of Physical Activity in the Early Years, Factors Influencing Physical Activity and Sedentary Behaviour in Childcare*) were logical. Considering outdoor time is a required component of all childcares in Canada, coupled with the knowledge that children accumulate the majority of their daily MVPA outdoors while attending childcare, [40] the high prioritization of *Outdoor Play* by both expert panels is reassuring and important to educate ECE students about. The introductory content area regarding benefits of physical activity stresses the need to provide ECE students with solid foundational knowledge of physical activity and sedentary behaviour. Further, overviewing the factors influencing children’s movement behaviours in the childcare environment was considered very important. Specifically, early childhood educator behaviours, known to influence children’s movement behaviours in childcare, [41] was highlighted as critical for targeted education. Taken together, the top-rated content areas represent topics in need of focus within training interventions for early childhood educators, and are pertinent to include in the e-Learning module for ECE students.

The moderate-to-strong inter-panel agreement, both in terms of content area mean score and rank-order, demonstrates general consensus regarding the importance of each topic for inclusion within the module. While select content areas were rated higher by one panel than the other (e.g., *Creation of Physical Activity/Screen-Viewing Policies* was favoured by the physical activity/sedentary behaviour experts, and *Risky Play* was favoured by the ECE experts), most content areas were similarly rated and ranked by both panels. Given the overarching goal of the TEACH study is to implement this e-Learning module in ECE post-secondary programs, it is critical that the content created for the module is pertinent to the ECE field. It is reassuring, then, that the large majority of ECE experts rated this training module as both in line with objectives of, and of added benefit to, the current post-secondary ECE curriculum. Hnatiuk and colleagues [42] stress the importance of tailoring early years physical activity interventions to community needs (in this case, lack of physical activity and sedentary behaviour training in the present ECE curriculum). With the overwhelming support of the ECE expert panel (nearly 100% of ECE experts reported this training was important for ECE students to receive), the creation of the e-Learning module using the content areas generated from this Delphi study is likely to be well-received by ECE programs within Canadian post-secondary institutions.

### Research implications and future directions

This research study has a number of important implications. First, the results of this study will be used to generate a physical activity and sedentary behaviour e-Learning course that is tailored specifically to ECE students, the first study globally to focus this training within early childhood educators’ pre-service education. Having educators who are well-trained in physical activity and sedentary behaviour will ensure children in childcare are provided sufficient movement opportunities daily, which is vital for their healthy development. Second, the recruitment of top international experts in the field to generate the content for the module ensures that this training covers the most important and relevant information for ECE students to promote healthy movement behaviours in childcare-based professions upon graduation. In addition, having a diverse panel of ECE experts review the content proposed by the physical activity/sedentary behaviour panel confirmed the applicability of this training to ECE, and will ease its receptivity by post-secondary ECE programs.

The implementation of the e-Learning course across Canada will shed light on whether this training is successful in ECE programs in multiple locations. In Canada, ECE curricula and professional accreditation standards are governed at the provincial/territorial level; as such, testing the effectiveness of this educational tool nationwide will determine the versatility of the e-Learning course to be implemented in multiple educational environments. If successful, the e-Learning course can be adapted (e.g., changing country-specific movement guidelines) for use in other countries, which would maximize the reach and global public health impact of this training. Given the global call for physical activity and sedentary behaviour training to be made available within early childhood educators’ pre-service schooling, [20, 28, 43] international collaborations are warranted to support this initiative.

### Limitations

Although this study has many strengths, including a high online survey response rate (53% for physical activity experts, 58% for ECE experts) and the use of the Delphi technique with two field-specific expert panels, it is not without limitations. First, the purposeful sampling method may have introduced selection bias. While efforts were made by the research team to overcome this bias (e.g., ensuring sufficient recruitment of international/provincial experts, allowing participants to suggest researchers to recruit), the selection of experts by the research team may have included experts with similar ideals and values regarding the importance of this training in ECE; this may limit the generalizability of the findings. Second, despite the anonymous nature of the online surveys, participants may have been subject to social desirability bias, as they may have felt that higher importance ratings were ‘expected’ of someone in their profession. Third, as is the case in any Delphi study, data gathered were based upon availability and the subjective opinion and expertise of participants.

## Conclusion

Using the Delphi method to identify and reach agreement on physical activity and sedentary behaviour-related topics to include in supplementary training for post-secondary ECE students provided a unique perspective on e-Learning module content development. The high importance ratings of all 19 content areas, coupled with the moderate-to-strong inter-panel agreement across these topics, suggest the need for this tailored education. Further, the agreement by the ECE expert panel regarding the appropriateness of incorporating this type of training within ECE programs demonstrated that there is a desire for physical activity and sedentary behaviour-related education at the post-secondary level, and that the addition of this content would support curriculum objectives and accreditation standards. Moving forward, creating an e-Learning module with evidence-based and expert-developed content, endorsed by those working in a wide range of ECE professions, will ensure that ECE graduates receive the necessary and most relevant education to be able to promote children’s healthy development of movement behaviours in childcare settings. Integrating such physical activity and sedentary behaviour training within ECE programs is a population-level approach to public health that has the potential to benefit a vast number of young children.

## Supplementary information

**Additional file 1.** Physical Activity Expert Survey 1. List of proposed content areas.

**Additional file 2.** Physical Activity Expert Survey 2. Importance ratings of content areas.

**Additional file 3.** Early Childhood Education Expert Survey. Importance ratings of content areas and additional proposed content.

## Data Availability

The dataset generated and analyzed during the present study is available from the corresponding author upon reasonable request.

## Abbreviations

ECE: Early Childhood Education
TEACH: Training EArly CHildhood educators in physical activity

## Footnotes

1 All mean scores presented as *M* ± *SD*

2 Five members of the panel did not respond to the second round of surveys.
