# Supplementary material for: Content development for a physical activity and sedentary behaviour e-learning module for early childhood education students: a Delphi study
Source: BMC Public Health. 2020 Oct 23;20:1600. doi: 10.1186/s12889-020-09670-w (PMC7584084; doi:10.1186/s12889-020-09670-w)
Supplement: Supplementary file 1 — Additional file 1. Physical Activity Expert Survey 1. List of proposed content areas. [file 12889_2020_9670_MOESM1_ESM.pdf]

1 - Creating a Physical Activity and Sedentary Behaviour e-Learning Module for ECE Students

Q1 Please enter your participant ID (sent to you in your initial invitation email)

____

Block 1

Q2 Please list your top 12 topics you believe should be covered in a Physical Activity and Sedentary Behaviour e-Learning Module for Early Childhood Education Students below, and provide a brief description to justify your topic choice. Please note these topics are not ranked.

Q3 Topic 1:

____

Q4 Topic 1 Justification:

____

Q5 Topic 2:

____

Q6 Topic 2 Justification:

____

Q7 Topic 3:

____

Q8 Topic 3 Justification:

____

Q9 Topic 4:

____

Q10 Topic 4 Justification:

____

Q11 Topic 5:

____

Q12 Topic 5 Justification:

____

Q13 Topic 6:

____

Q14 Topic 6 Justification:

____

Q15 Topic 7:

____

Q16 Topic 7 Justification:

____

Q17 Topic 8:

____

Q18 Topic 8 Justification:

____

Q19 Topic 9:

____

Q20 Topic 9 Justification:

____

Q21 Topic 10:

____

Q22 Topic 10 Justification:

____

Q23 Topic 11:

____

Q24 Topic 11 Justification:

____

Q25 Topic 12:

____

Q26 Topic 12 Justification:

____
