# Supplementary material for: Content development for a physical activity and sedentary behaviour e-learning module for early childhood education students: a Delphi study
Source: BMC Public Health. 2020 Oct 23;20:1600. doi: 10.1186/s12889-020-09670-w (PMC7584084; doi:10.1186/s12889-020-09670-w)
Supplement: Supplementary file 3 — Additional file 3. Early Childhood Education Expert Survey. Importance ratings of content areas and additional proposed content. [file 12889_2020_9670_MOESM3_ESM.pdf]

3 - Creating a Physical Activity and Sedentary Behaviour e-Learning Module for ECE Students

Start of Block: Block 7

Q4 Please enter your participant ID (sent to you in your initial invitation email)

____

End of Block: Block 1

Start of Block: Block 10

Q36 What is your current position/role at your organization/institution?

____

Q37 How many years of experience do you have working in the Early Childhood Education field?

____

End of Block: Block 10

Start of Block: Default Question Block

Q2 Physical activity and sedentary behaviour-related topics were generated by a panel of physical activity and sedentary behaviour experts in early childhood research. A total of 19 content areas were suggested for inclusion in the e-Learning module, and these have been assembled into 4 modules:

Module 1: An Introduction to Physical Activity and Sedentary Behaviour in the Early Years Module 2: Physical Activity and Sedentary Behaviour in the Childcare Environment Module 3: How to Promote Physical Activity and Minimize Sedentary Time

in Childcare Module 4: Training, Resources, and Practical Examples of Activities

Q5 Please rank your perceived importance of the following list of topics for inclusion in a Physical Activity and Sedentary Behaviour e-Learning Module for Early Childhood Education students. Each content area is listed along with a description of what would be covered in this section of the e-Learning module.

Q12 **Module #1 - An Introduction to Physical Activity and Sedentary Behaviour in the Early Years**

Q1
Content Area #1 - Defining Physical Activity and Sedentary Behaviour
• What is physical activity, and what does this behaviour look like in infants (<1y), toddlers (1-2y), and preschoolers (3-4y)?
o What are the intensities of movement behaviours? (i.e., sedentary, light, moderate, and vigorous activity)
o Structured vs. unstructured physical activity
o What is active play?
• What is sedentary behaviour, and what does this behaviour look like in infants, toddlers, and preschoolers?
• What is screen-viewing?
o Active vs. passive screen-viewing
o Media usage in childcare – appropriate for educational purposes?

	Unimportant (1)	Of Little Importance (2)	Moderately Important (3)	Important (4)	Very Important (5)
Defining Physical Activity and Sedentary Behaviour (1)	o	o	o	o	o

Q38 Do you have any comments or feedback on this content area?

____

Q6

Content Area #2 - The Canadian 24-Hour Movement Guidelines for the Early Years (<5 years)

• How much light, and moderate-to vigorous-intensity physical activity should young children engage in each day?
• How much continuous sitting time should young children be limited to?
• How much screen-viewing should young children be limited do each day?
• How can these guidelines be adapted to the childcare day?

	Unimportant (1)	Of Little Importance (2)	Moderately Important (3)	Important (4)	Very Important (5)
The Canadian 24-Hour Movement Guidelines for the Early Years (1)	○	○	○	○	○

Q39 Do you have any comments or feedback on this content area?

____

Q7

Content Area #3 - Prevalence of Physical Activity, Sedentary Behaviour, and Screen-Viewing Among Young Children

• Overall prevalence rates, and in various childcare settings (home-based childcare vs. centre-based childcare vs. full-day kindergarten)
• How do these compare to guidelines?

• Sociodemographic differences in movement behaviours

	Unimportant (1)	Of Little Importance (2)	Moderately Important (3)	Important (4)	Very Important (5)
Prevalence of Physical Activity, Sedentary Behaviour, and Screen-Viewing Among Young Children (1)	○	○	○	○	○

Q40 Do you have any comments or feedback on this content area?

____

Q8
Content Area #4 - What are the Benefits of Physical Activity in the Early Years?
• Links with improved physical, psychosocial, and cognitive development (e.g., improved bone and skeletal health, weight status, brain development, emotional regulation)
• Importance of the early years for establishing physical activity habits that set the foundation for an active childhood, adolescence, and adulthood

	Unimportant (1)	Of Little Importance (2)	Moderately Important (3)	Important (4)	Very Important (5)
What are the Benefits of Physical Activity in the Early Years? (1)	○	○	○	○	○

Q41 Do you have any comments or feedback on this content area?

____

Q9 **Content Area #5 - Physical Literacy and Fundamental Movement Skills**
· Fundamental movement skills and sport skills · APPLE Model – Active Play and Physical Literacy Every day · Building confidence and competence in a variety of physical activity settings (via mastery experiences, vicarious experiences, verbal persuasion)
· Importance of physical literacy for lifelong participation in physical activities

	Unimportant (1)	Of Little Importance (2)	Moderately Important (3)	Important (4)	Very Important (5)
Physical Literacy and Fundamental Movement Skills (1)	○	○	○	○	○

Q42 Do you have any comments or feedback on this content area?

____

Q10
Content Area #6 - What are the Risks of Excessive Sedentary Behaviour, Particularly Screen-Viewing?
• Independent of physical activity, links with physical health (e.g., weight status), behaviour, cognitive development (including language development), irregular sleep patterns
• Establishing sedentary behaviour and screen-viewing habits that set the foundation for a

healthy future

	Unimportant (1)	Of Little Importance (2)	Moderately Important (3)	Important (4)	Very Important (5)
What are the Risks of Excessive Sedentary Behaviour, Particularly Screen-Viewing? (1)	○	○	○	○	○

Q43 Do you have any comments or feedback on this content area?

____

End of Block: Default Question Block

Start of Block: Block 2

Q11 **Module #2 - Physical Activity and Sedentary Behaviour in the Childcare Environment**

Q13
Content Area #7 - Factors Influencing Physical Activity and Sedentary Behaviour in Childcare
• ECEs are important influences on young children’s movement behaviours (in terms of programming, role modeling, and training in physical activity)
• Presence/size of indoor and outdoor play areas
• Fixed and portable play equipment
• Scheduling of outdoor time

• Physical activity and screen-viewing policies

	Unimportant (1)	Of Little Importance (2)	Moderately Important (3)	Important (4)	Very Important (5)
Factors Influencing Physical Activity and Sedentary Behaviour in Childcare (1)	○	○	○	○	○

Q44 Do you have any comments or feedback on this content area?

____

Q14

Content Area #8 - Outdoor Play

• Importance of outdoor play for physical, psychosocial, and cognitive health (e.g., increased physical activity, improved mood and creativity)

• Outdoor play in various climates (cold, rain, snow, extreme heat) and solutions if outdoor time is not an option

• How to make the most out of outdoor space for physical activity

	Unimportant (1)	Of Little Importance (2)	Moderately Important (3)	Important (4)	Very Important (5)
Outdoor Play (1)	○	○	○	○	○

Q45 Do you have any comments or feedback on this content area?

____

Q15

Content Area #9 - Risky Play

• Importance of risky/adventurous play (building confidence through appropriate challenge)
• Difference between risky and dangerous/hazardous play
• How to encourage risky play among young children
• Cost/benefit analysis of engaging in risky play

	Unimportant (11)	Of Little Importance (12)	Moderately Important (13)	Important (14)	Very Important (15)
Risky Play (1)	○	○	○	○	○

Q46 Do you have any comments or feedback on this content area?

____

End of Block: Block 2

Start of Block: Block 3

Q16 **Module #3 - How to Promote Physical Activity and Minimize Sedentary/Screen Time in Childcare**

Q17

Content Area #10 - Monitor Physical Activity and Sedentary Time in Your Classroom

• How are movement behaviours measured in research?
• How can movement behaviours be monitored by ECEs?
• Models of behaviour change to inform physical activity promotion strategies
• How can ECEs create goals and track progress after implementing physical activity-promoting changes?

	Unimportant (1)	Of Little Importance (2)	Moderately Important (3)	Important (4)	Very Important (5)
Monitor Physical Activity and Sedentary Time in Your Classroom (1)	○	○	○	○	○

Q47 Do you have any comments or feedback on this content area?

____

Q18
Content Area #11 - Become a Role Model and Champion for Physical Activity
• Benefits of role modeling and co-participation
• Actively participating in outdoor play (not simply supervising)

	Unimportant (1)	Of Little Importance (2)	Moderately Important (3)	Important (4)	Very Important (5)
Become a Role Model and Champion for Physical Activity (1)	○	○	○	○	○

Q48 Do you have any comments or feedback on this content area?

____

Q19

Content Area #12 - Promote Physical Activity and Minimize Sedentary Time through Instruction and Interaction

• Avoid withholding physical activity and outdoor time as punishment, or using screens as a reward
• Involve children in daily activities (hanging coats, clearing the table, etc.)
• Notice individual differences and learn how each child responds to forms of encouragement to be active
• How to facilitate active play

	Unimportant (1)	Of Little Importance (2)	Moderately Important (3)	Important (4)	Very Important (5)
Promote Physical Activity and Minimize Sedentary Time through Instruction and Interaction (1)	○	○	○	○	○

Q49 Do you have any comments or feedback on this content area?

____

Q20

Content Area #13 - Program Time for Physical Activity and Active Breaks to Limit Sitting Time

• How to design curriculum to be supportive of physical activity and minimize sitting time
• Teacher-led physical activity
• Developing physical activity opportunities for children of all abilities
• Scheduling indoor free play time
• How to incorporate muscle and bone-strengthening activities into programming
• Programming active breaks between sedentary tasks to break up prolonged sitting

• How to minimize sedentary behaviour during transition times

	Unimportant (1)	Of Little Importance (2)	Moderately Important (3)	Important (4)	Very Important (5)
Program Time for Physical Activity and Active Breaks to Limit Sitting Time (1)	○	○	○	○	○

Q50 Do you have any comments or feedback on this content area?

____

Q21
Content Area #14 - Incorporate Physical Activity into Other Educational Objectives
• How to integrate physical activity into other curriculum areas and typically sedentary activities (e.g., reading circles, arts and crafts)

	Unimportant (1)	Of Little Importance (2)	Moderately Important (3)	Important (4)	Very Important (5)
Incorporate Physical Activity into Other Educational Objectives (1)	○	○	○	○	○

Q51 Do you have any comments or feedback on this content area?

____

Q22

Content Area #15 - Create and Make Use of Environments to be Supportive of Physical Activity

- How to set up your classroom to promote movement
- Making use of limited spaces/resources (moving furniture, using hallways)

	Unimportant (1)	Of Little Importance (2)	Moderately Important (3)	Important (4)	Very Important (5)
Create and Make Use of Environments to be Supportive of Physical Activity (1)	○	○	○	○	○

Q52 Do you have any comments or feedback on this content area?

____

Q23

Content Area #16 - Suggest the Creation of Physical Activity and Screen-Viewing Policies at your Centre

• Having a written physical activity and screen-viewing policy will aid in achieving goals to increase physical activity and minimize screen-viewing

• This will help parents understand that the centre prioritizes their child's health

	Unimportant (1)	Of Little Importance (2)	Moderately Important (3)	Important (4)	Very Important (5)
Suggest the Creation of Physical Activity and Screen-Viewing Policies at your Centre (1)	o	o	o	o	o

Q53 Do you have any comments or feedback on this content area?

____

Q24

Content Area #17 - Get Parents/Guardians on Board!

• Communicate with parents about the importance of physical activity and minimizing screen-viewing in early childhood

• Communicate with parents about their child's movement behaviours at childcare, and how they can support progress at home

	Unimportant (1)	Of Little Importance (2)	Moderately Important (3)	Important (4)	Very Important (5)
Get Parents/Guardians on Board! (1)	o	o	o	o	o

Q54 Do you have any comments or feedback on this content area?

____

End of Block: Block 3

Start of Block: Block 4

Q25 **Module #4 - Training, Resources, and Practical Examples of Activities**

Q26
Content Area #18 - Resources and Professional Development
• Importance of background training in physical activity and sedentary behaviour, and on-going professional development
• Many childcare organizations provide professional development workshops for ECEs
• Examples of professional development in physical activity and health (e.g., HighFIVE, Physical Literacy Instructor Program)
• Resources – Sport for Life, Active for Life, OPHEA, Healthy Kids Healthy Future
• Resources – Colleagues, social media (e.g., Pinterest)

	Unimportant (1)	Of Little Importance (2)	Moderately Important (3)	Important (4)	Very Important (5)
Resources and Professional Development (1)	○	○	○	○	○

Q55 Do you have any comments or feedback on this content area?

____

Q27
Content Area #19 - Example Activities

• Video examples of teacher-led physical activities and active breaks

	Unimportant (1)	Of Little Importance (2)	Moderately Important (3)	Important (4)	Very Important (5)
Example Activities (1)	○	○	○	○	○

Q56 Do you have any comments or feedback on this content area?

____

End of Block: Block 4

Start of Block: Block 5

Q31 Are there any topics that you believe were missed and should be included in the e-Learning module? If so, please describe:

____

Q57 Does the proposed Physical Activity and Sedentary Behaviour e-Learning Module for Early Childhood Education students:

	Strongly disagree (8)	Disagree (9)	Neither agree nor disagree (10)	Agree (11)	Strongly agree (12)
Align with ECE curriculum objectives? (1)	○	○	○	○	○
Complement the current ECE curriculum? (3)	○	○	○	○	○
Align with ECE accreditation standards/vocational learning outcomes? (2)	○	○	○	○	○

Q59 Please provide any comments or feedback you have related to the above:

____

Q58 How important do you believe this type of training is for Early Childhood Education students?

○ Unimportant (1)

○ Of Little Importance (2)

○ Moderately Important (3)

○ Important (4)

○ Very Important (5)

End of Block: Block 5

Start of Block: Block 6

Q29 Please rank order the content areas proposed by your perceived ***importance of their inclusion in the e-Learning module***:

______ Defining Physical Activity and Sedentary Behaviour (1)
______ The Canadian 24-Hour Movement Guidelines for the Early Years (2)
______ Prevalence of Physical Activity, Sedentary Behaviour, and Screen-Viewing Among Young Children (3)
______ What are the Benefits of Physical Activity in the Early Years? (4)
______ Physical Literacy and Fundamental Movement Skills (5)
______ What are the Risks of Sedentary Behaviour, Particularly Screen-Viewing? (6)
______ Factors Influencing Physical Activity and Sedentary Behaviour in Childcare (7)
______ Outdoor Play (8)
______ Risky Play (9)
______ Monitor Physical Activity and Sedentary Time in Your Classroom (10)
______ Become a Role Model and Champion for Physical Activity (11)
______ Promote Physical Activity and Minimize Sedentary Time through Instruction and Interaction (12)
______ Program Time for Physical Activity and Active Breaks to Limit Sitting Time (13)
______ Incorporate Physical Activity into Other Educational Objectives (14)
______ Create and Make Use of Environments to be Supportive of Physical Activity (15)
______ Suggest the Creation of Physical Activity and Screen-Viewing Policies at Your Centre (16)
______ Get Parents/Guardians on Board! (17)
______ Resources and Professional Development (18)
______ Example Activities (19)

Q30 Please rank order the content areas proposed by ***the order you would introduce them in the e-Learning module***:

______ Defining Physical Activity and Sedentary Behaviour (1)
______ The Canadian 24-Hour Movement Guidelines for the Early Years (2)
______ Prevalence of Physical Activity, Sedentary Behaviour, and Screen-Viewing Among Young Children (3)
______ What are the Benefits of Physical Activity in the Early Years? (4)
______ Physical Literacy and Fundamental Movement Skills (5)
______ What are the Risks of Sedentary Behaviour, Particularly Screen-Viewing? (6)
______ Factors Influencing Physical Activity and Sedentary Behaviour in Childcare (7)
______ Outdoor Play (8)
______ Risky Play (9)
______ Monitor Physical Activity and Sedentary Time in Your Classroom (10)
______ Become a Role Model and Champion for Physical Activity (11)
______ Promote Physical Activity and Minimize Sedentary Time through Instruction and Interaction (12)
______ Program Time for Physical Activity and Active Breaks to Limit Sitting Time (13)
______ Incorporate Physical Activity into Other Educational Objectives (14)
______ Create and Make Use of Environments to be Supportive of Physical Activity (15)
______ Suggest the Creation of Physical Activity and Screen-Viewing Policies at Your Centre (16)
______ Get Parents/Guardians on Board! (17)
______ Resources and Professional Development (18)
______ Example Activities (19)

Page Break

Q32 The Physical Activity and Sedentary Behaviour e-Learning module will have roughly 6 hours of content. **Please drag the module sections to the time allotment you perceive should be dedicated to that module section**:

<1 hour	1 - 1.5 hours	1.5 - 2 hours	>2 hours
_______ Module 1: An Introduction to Physical Activity and Sedentary Behaviour in the Early Years (1)	_______ Module 1: An Introduction to Physical Activity and Sedentary Behaviour in the Early Years (1)	_______ Module 1: An Introduction to Physical Activity and Sedentary Behaviour in the Early Years (1)	_______ Module 1: An Introduction to Physical Activity and Sedentary Behaviour in the Early Years (1)
_______ Module 2: Physical Activity and Sedentary Behaviour in the Childcare Environment (2)	_______ Module 2: Physical Activity and Sedentary Behaviour in the Childcare Environment (2)	_______ Module 2: Physical Activity and Sedentary Behaviour in the Childcare Environment (2)	_______ Module 2: Physical Activity and Sedentary Behaviour in the Childcare Environment (2)
_______ Module 3: How to Promote Physical Activity and Minimize Sedentary/Screen Time in Childcare (3)	_______ Module 3: How to Promote Physical Activity and Minimize Sedentary/Screen Time in Childcare (3)	_______ Module 3: How to Promote Physical Activity and Minimize Sedentary/Screen Time in Childcare (3)	_______ Module 3: How to Promote Physical Activity and Minimize Sedentary/Screen Time in Childcare (3)
_______ Module 4: Training, Resources, and Example Activities (4)	_______ Module 4: Training, Resources, and Example Activities (4)	_______ Module 4: Training, Resources, and Example Activities (4)	_______ Module 4: Training, Resources, and Example Activities (4)

End of Block: Block 6

Start of Block: Block 9

Q35 Please list the name(s) of any ECE professionals you believe should be included on the ECE expert panel for the creation of this module:

____

End of Block: Block 9

Start of Block: Block 8

Q34 If you wish to receive a copy of the study results, use the following link and enter your email:
https://uwo.eu.qualtrics.com/jfe/form/SV_1NyBSEg2ZzJQp5H

Thanks for your participation! Please submit this page with the arrow below.

End of Block: Block 8
